# Pre-Pregnancy Counselling for Women with Rheumatoid Arthritis: A Guide on Risks, Evaluations, and Multidisciplinary Approaches

**DOI:** 10.3390/jcm14010114

**Published:** 2024-12-28

**Authors:** Ioana Cristina Saulescu, Anca Maria Panaitescu, Nicolae Gică, Elena Grădinaru, Daniela Opris-Belinski

**Affiliations:** 1 Department of Internal Medicine and Rheumatology, Carol Davila University of Medicine and Pharmacy, 050474 Bucharest, Romania; ioana.saulescu@umfcd.ro (I.C.S.);; 2 Department of Internal Medicine and Rheumatology, Sfanta Maria Hospital, 011172 Bucharest, Romania; 3 Filantropia Clinical of Obstetrics and Gynecology Hospital, 011160 Bucharest, Romania

**Keywords:** rheumatoid arthritis, pre-pregnancy, medication adjustment

## Abstract

This paper explores the essential role of pre-pregnancy counselling for women with rheumatoid arthritis (RA), focusing on minimising risks and optimising pregnancy outcomes. RA, a prevalent inflammatory arthritis with onset during childbearing years, necessitates targeted preconception counselling to manage disease activity and comorbidities effectively. The counselling ensures medication compatibility and planning around disease flares, and it involves a multidisciplinary team comprising rheumatologists, obstetricians, and other specialists to develop individualised care plans. This literature review highlights the challenges women with RA face, including prolonged time to pregnancy, increased risks during pregnancy, such as hypertension and preeclampsia, and potential fertility issues related to medication and disease activity. Emphasis is placed on the importance of assessing autoantibody presence and managing specific joint involvements that may affect anaesthetic procedures during pregnancy. This paper underscores the importance of timing conception during periods of low disease activity and adopting a “Treat-to-Target” approach using acceptable medications to maintain disease remission. This study calls for routine family planning discussions and preconception evaluations to address reproductive health and treatment plans, thereby supporting women with RA in achieving favourable pregnancy outcomes comparable to the general population. The multidisciplinary approach and regular counselling are critical to navigating the complexities of RA and pregnancy successfully.

## 1. Introduction

Pre-pregnancy counselling has the potential role of reducing adverse outcomes of a pregnancy from the woman’s, foetus’s, and neonate’s point of view, helping the future mother to reduce risks related to modifiable risk factors (smoking, drinking alcohol, etc.) or to optimise health when a chronic condition/medication is in line. This implies that pre-pregnancy counselling should be addressed to any woman during the childbearing years whether she is or is not planning a pregnancy, and this should occur as often as possible to increase their knowledge and maximise the outcome [1].

Rheumatoid arthritis (RA) is a chronic condition, being one of the most common types of inflammatory arthritis (IA) with common onset during childbearing age, characterised by joint and sometimes extra-skeletal involvement [2]. Appropriate preconception counselling is critical for women with RA regarding disease activity, associated comorbidities, as well as therapy that may have an impact on pregnancy outcomes. A general assessment of risk in patients considering pregnancy should include the identification of serious organ damage that might affect the ability to safely carry a pregnancy, evaluation of disease activity, and serologic screening to identify possible autoantibodies associated with adverse foetal outcomes, with mandatory revision of medications for compatibility with pregnancy. Advanced pregnancy planning also facilitates the ability to review and adjust medications for compatibility with pregnancy [3]. However, considering that the natural history of RA involves a relapsing–remitting course, avoiding conception during periods of disease flares is important for both maternal health and foetal outcomes. Thus, family planning supports periodic preconception counselling as an essential part of care for all female patients of reproductive age, not only for women expressing the desire to become pregnant [4].

All of the steps mentioned above should be optimised by a multidisciplinary team formed by rheumatologists, obstetricians, general practitioners, or other specialists who need to be involved. Close communication between them is necessary for developing individualised plans not only to treat active disease but also to maintain disease remission during the preconception phase, pregnancy, and postpartum with an approved treatment plan, thus increasing the chance for an optimal outcome [5].

## 2. Materials and Methods

We conducted a literature review of medical databases, including PubMed, Google Scholar, and Cochrane Controlled Trials Register, for relevant information on the impact of pregnancy on female RA patients, preconception evaluation, and the impact of the mentioned disorder on fertility, pregnancy, and newborns. The keywords used for the search included preconception, pregnancy, rheumatoid arthritis, autoimmune disease, and inflammatory arthritis. Titles and abstracts from 2000 to 2024 were identified. When an abstract was selected, the full-text article was evaluated and, if eligible, selected to be used. Reference lists of this articles were also checked for additional records.

**Figure 1:**
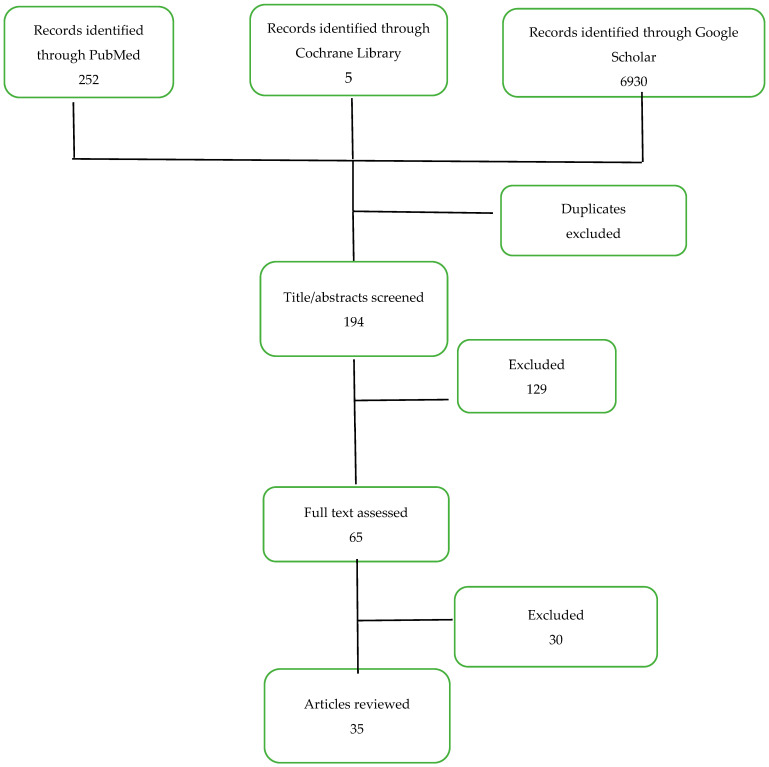
Results of systematic search and selection process.

Articles were considered eligible if they covered preconception in RA or IA, guidelines for the management of pregnancy in this particular patient, or treatment recommendations. Articles were excluded in the case of editorial papers, articles not covering RA or IA, when the language used was other than English, or when the content was not relevant to the subject. This process is described in Figure 1.

## 3. Results

### 3.1. Patient Perspective

In the last 10 years, there has been a steady increase in the number of pregnancies in patients with RA [6], which is mostly related to better knowledge of both future parents and caregiver providers. However, when evaluating the parity of women with inflammatory arthritis compared to controls using the Norwegian Population Registry, it was more likely for women with inflammatory arthritis to remain childless compared to controls [5]. Family size is reduced for women with RA [7,8], which is related to patient choice, the disease itself, or medication [8,9].

Women of reproductive age living with RA express a lot of concerns when prospecting parenthood due to physical impairment related to disease that can impact pregnancy or the ability to carry a child due to fear of passing the disease onto the child, exposure to medication needed for disease control, or possible progression of the disease during pregnancy [9,10]. Smaller family size was associated with younger age at diagnosis, especially if they were diagnosed with RA before having their first children [7].

Ackerman et al. conducted a study trying to identify the information needed by women with RA who are prospecting motherhood. In total, 27 women with RA who were pregnant in the last 5 years, currently pregnant, or contemplating pregnancy were interviewed. The results showed that the rheumatologist was the principal source of information, especially from the treatment perspective during pregnancy, but most of the participants expressed the need for sharing information and experience with other women to help them lower the stress related to medication exposure and learn practical tips needed to cope with daily challenges during and after pregnancy [9].

Although women with RA have frequent visits to rheumatologists, a study conducted by Chakravarty about family planning in patients with inflammatory diseases, including RA, found that these patients prefer a gynaecologist as the main provider of preconception counselling, while the treating physician should offer a solution only related to medication [11]. Periodic referral of women with RA to the gynaecologist to discuss subjects related to reproductive health has to become part of the rheumatological evaluation.

Women with RA who are trying to conceive need prolonged time when compared with women without RA, suggesting a reduction in fertility [12]. Time to pregnancy (TTP) is considered a reliable measure of fertility, with subfertility being associated with a TTP greater than 12 months. When compared with the general population, women with RA have a higher percentage of subfertility of 25–42% versus 9% [8,13].

Impaired fertility might be related to different factors like medication or disease activity. Preconception use of nonsteroidal anti-inflammatory drugs (NSAIDs), especially selective cyclooxygenase 2 inhibitors, will inhibit prostaglandin production and subsequently negatively impact ovulation or nidation [8,14]. Higher doses of corticosteroids—greater than 7.5 mg of Prednisone equivalent/day—will suppress the hypothalamic–pituitary axis and have a direct negative effect on the endometrium and ovarian function [8,13]. Although not contraindicated during conception, discussion with the future mother about these effects and encouraging her to obtain and maintain proper control of the disease with limited or no use of NSAIDs or a higher corticosteroid dose than permitted will increase the chance of obtaining a pregnancy without anxiety related to a prolonged TTP.

Brouwer et al. prospectively followed 245 women with RA (PARA study) who were included preconceptionally or during the first trimester. Longer TTP was associated with nulliparity, age, and higher disease activity evaluated by the classical score, the Disease Activity Score 28 (DAS 28), with no association with other factors like seropositive status for Rheumatoid Factor (RF) or anti-citrullinated peptide antibodies (ACPA), current use of Sulfasalazine (SSZ), or past use of Methotrexate (MTX) [15].

Future mothers carrying an RA diagnosis should be informed during the pre-pregnancy evaluation about fertility issues, with an emphasis on choosing the right time when the disease is well-controlled under approved medication. There are data showing that in 41% of subfertile women with RA, no specific cause for subfertility can be found compared with 8–28% in the general population, indicating that disease-related factors are the ones that prolonged TTP [16]. Presenting these data during preconception counselling should help future mothers understand the importance of proper preparation for pregnancy.

Another important aspect that should be discussed with female patients, especially if they express a desire for future pregnancy, is the early menopause observed in some women with RA, possibly related to a smaller ovarian reserve. Anti-Mullerian hormone, a marker for ovarian reserve, was found to be reduced in established disease compared with healthy controls, but it was normal in early disease, pointing again to RA or medication as a possible cause [17]. This is important information that should be shared with all fertile women at the moment of RA diagnosis to try to raise awareness of the importance of good judgment when planning a family.

Family planning/preconception counselling should become routine when female RA patients are evaluated by the treating physicians. Couples’ referral to a specialised obstetrician to assess possible fertility problems and proceed to a thorough evaluation to optimise the outcome is a step that needs to be implemented more often. Preservation of emotional well-being is very important; this is why discussing with RA patients the existence of alternative pathways like ART will maintain an optimistic approach to future pregnancy [16].

### 3.2. Preconception Counselling: The Doctor’s Perspective

When Is the Right Time for an RA Patient to Consider a Future Pregnancy?

Historically, RA was one of the most debilitating IAs affecting the young adult population, sometimes before being able to accomplish their dreams related to family or family size. Taking into account limited treatment options, late diagnosis, and low compliance, back then, a lot of patients accumulated irreversible joint damage or extra-skeletal comorbidities, contributing to the belief that women with RA would not be able to successfully carry a pregnancy or take care of a baby [18]. This has changed nowadays with the newly available treatments and with the Treat-To-Target concepts, permitting rapid and efficient control of active disease, with an impact on disease activity, functional articular status, and disease- or treatment-related complications [19]. Being able to have a nearly normal life with the help of proper medication, the perception of pregnancy has changed both for the RA patient but also from the physician’s point of view.

The European Alliance of Associations for Rheumatology (EULAR) and The American College of Rheumatology (ACR) have issued recommendations on how to use antirheumatic drugs before, during, and after pregnancy, but also about reproductive health in rheumatic and musculoskeletal disease, including RA [20,21]. It is postulated now that the best pregnancy outcome is obtained if conception takes place during stable, inactive, or low disease activity under permitted medication, which usually must continue during the 9 months to avoid new flares [21,22], reinforcing the idea that preconception counselling should start as of the RA diagnosis in all potential future parents and periodically repeated as long as needed.

#### 3.2.1. RA Activity

A traditional approach to the categorisation of autoimmune diseases (AIDs) is based on the predominant cytokine profile characteristic of two major T-helper cell subsets: Th1 and Th2. Therefore, during pregnancy and shortly after delivery, there is a decreased Th1/Th2 cytokine ratio, and diseases with Th1 predominance, like RA, tend to ameliorate during pregnancy but flare in the postpartum period [4]. The explanation is related to the fact that the major phase of foetal development and growth is characterised by an anti-inflammatory and Th2-type immune microenvironment [23,24]. Unfortunately, this is not the case for all women with RA. A recent metanalysis, published in 2019 showed that the improvement rate during pregnancy is about 60% [25], suggesting the need for continuous proper monitoring and medication. Explanations for the decreasing improvement rate (this rate was 75–95% in older studies, [13]) are the absence of standardised measures of RA activity in older studies, the appearance of Treat-to-Target concepts with beneficial effects in controlling disease activity, and existing recommendations on when to pursue a pregnancy for patients with an AID [8,21]. Nowadays, even patients with severe RA can better control the disease, such as a shift in the possibility of a pregnancy regardless of RA severity. Aggressive arthritis can be treated now with adequate medication, not only classical disease-modifying antirheumatics drugs (DMARDs), making pursuing a pregnancy a reality, with the increased need for treatment during gestation [26,27].

Disease evolution during a future pregnancy should be discussed with all women with RA who express the desire to conceive, emphasising the importance of regular check-ups with obstetricians and treating rheumatology physicians. The existence of a possible flare during the 9 months exists, although the probability is much lower for women who start the pregnancy during a remission/low disease activity (LDA) period [21]. Physicians should address this possibility, assuring her of the utility of adequate, properly chosen treatment to avoid the detrimental effect of active disease on foetal health. Convincing a future mother to take medication during pregnancy might be very challenging because of the popular idea that this is completely forbidden and harmful to the baby. Presenting data about the health and growth of children exposed in utero to different medications will help increase compliance and reduce future mother stress [28].

An important aspect to discuss at the preconception evaluation is the importance of the right time to conceive regarding disease activity, accepted medication, and specific comorbidities. Data accumulated, especially from registries, showed that pregnancy outcomes in RA women are slightly less favourable compared with the general population when there is active disease before and during pregnancy, but no differences from the general population were found in stable, inactive RA [29,30,31].

From the mother’s point of view regarding risk, pregnant women with RA have an increased risk for hypertension and preeclampsia when compared with the general population [13], and this should be discussed during preconception evaluation. Future mothers must be advised to regularly check their blood pressure and complete anamnesis, including their past medical and family history, such as past obstetrical events. There is also an increased risk for intrauterine growth restriction and infants small for gestational age, premature rupture of the membrane, and antepartum haemorrhage [13,29]. Preterm delivery (before 37 weeks) is more frequent in RA pregnancy, which is related to exposure to Prednisone and severe, active disease [13]. The Caesarean section rate is higher with higher disease activity during pregnancy [32,33]. There are conflicting results about miscarriages, with one study from the Norwegian registry describing an increased risk, possibly related to the inclusion of both planned and unplanned pregnancies, the last with high exposure to Methotrexate [13]. Presenting these data during preconception counselling will help convince the RA women about the importance of starting pregnancy when the disease is stable and well-controlled under accepted medication.

Measuring tools for disease activity in AID are sometimes complex and difficult to use in clinical practice, but this is not the case for RA, which only involves evaluation of joint involvement. Among the different scores used in RA, Disease Activity Score 28 (DAS 28) is very easy to use, permits the grading of disease activity, and measures improvement. It is used by EULAR and ACR for treatment recommendations. It is a composite score that evaluates 28 joints (tender and swollen), an inflammatory marker (Erythrocyte Sedimentation Rate (ESR) or C-Reactive Protein (CRP)), and Patient Global Health based on a visual analogue scale. Although the formula is complex, there are practical online calculators, and the results vary from 0 to 10. A change of at least 0.6 is considered a response when treatment is evaluated [34]. Data obtained from clinical trials demonstrated that DAS 28-CRP is lower than DAS 28-ESR, and this is reflected by the cut-off used to define disease status, as shown in Table 1. Both EULAR and ACR guidelines for treatment mention only DAS 28 without specifying whether ESR or CRP should be used. Moreover, these values are often used interchangeably both in clinical trials and in daily practice, using as the cut-off the value for DAS 28-ESR [35].

Standardised evaluation with the DAS 28 score helped to better characterise the trend in the evolution of RA during pregnancy, although there are limitations in using conventional measures of disease activity in pregnancy, as these measures can be confounded by symptoms related to pregnancy itself. Nevertheless, in a comparison of different disease activity scoring tools in pregnant women with RA versus healthy controls, the DAS28 calculated with CRP (DAS28-CRP) and without assessment of global health (GH) performed the best in pregnant RA patients [32]. The Pregnancy-Induced Amelioration in Rheumatoid Arthritis (PARA) study was a prospective study (performed from 2002 to 2010) that followed women with RA from preconception or early pregnancy to post-partum, helping to better characterise RA’s evolution during pregnancy. Until then, it was assumed that all RA patients would go into a remission state during pregnancy independent of treatment (or lack of treatment). Application of validated disease activity indices of DAS 28 confirmed that only 20–40% of patients with RA will achieve remission by the third trimester, 50% may be considered to have low disease activity, and nearly 20% will have worse or moderate to high disease activity and may require further therapeutic intervention. In this cohort, many women experienced postpartum flares preventing them from taking care of themselves and their infants [32,36]. Preparing future mothers for different scenarios will increase awareness about the need for proper evaluation before, during, and after pregnancy and adequate therapeutic decisions.

According to the most recent recommendation, pregnancy can be pursued if women with RA are in remission or have low disease activity for at least 6 months [21].

The Preconception Counseling in Active RA (Pre CARA) is an ongoing observational study (started in 2011) performed in a tertiary clinic specialised in autoimmune disorders and pregnancy (Erasmus MC, Rotterdam) that includes patients before they become pregnant and follows them every 3 months before conception and during pregnancy and at 6, 12, and 26 weeks postpartum. In this study, a Treat-to-Target (T2T) approach was considered for RA women preparing to conceive using an accepted combination of DMARDs, low-dose Prednisone, and Tumor Necrosis factor (TNF) inhibitors if needed. This T2T approach showed that the ACR recommendation [21] is attainable, as more than 80% of patients from this cohort entered pregnancy with LDA that was maintained throughout all 9 months and postpartum [37].

If patients have ongoing active disease, then the doctor should adjust the therapeutic scheme and must advise the patient to postpone the pregnancy and to use appropriate contraception until the next re-evaluation [21,38]. The ACR recommendation for women with an AID other than Systemic Lupus Erythematosus is to use effective contraceptive methods (for example, hormonal contraceptives or intrauterine devices (IUDs)) against less efficient methods or no contraception. The lowest failure rates are associated with IUDs and subdermal progestin implants (long-acting reversible contraception), these being highly recommended [21].

#### 3.2.2. Seropositive Versus Seronegative RA

Apart from disease activity, RA patients are characterised by autoantibody presence: Rheumatoid Factor (RF) and anti-citrullinated protein antibody (ACPA). Their titre is not influenced by pregnancy, but their presence [seropositivity], especially in high titres, is associated with the risk for more active disease during pregnancy, and seronegative patients are more likely to improve [8,39]. Preconception evaluation should assess the presence or absence of these autoantibodies, which are useful as prognostic factors.

#### 3.2.3. Extra-Skeletal RA Manifestations

Extra-skeletal (eye, skin, lung, heart, kidney, nervous system, salivary glands, or bone marrow) involvement in RA is associated with more severe, usually seropositive disease, being found in up to 40% of cases [40,41]. Other risk factors are age, presence of antinuclear antibodies apart from RF or ACPA, disease duration, early disability, and smoking. There are some reports that extraarticular features like rheumatoid nodules, vasculitis, or secondary Sjogren disease have been less frequently found in RA patients since the mid-1990s, which is probably related to better, targeted treatments [40,42].

Periodic evaluation for systemic manifestations must be part of RA patients’ consultations. Some of them are clinically silent, and lab, functional, or imaging tests should be performed yearly, allowing for early diagnosis and management. Because risk increases with age and disease duration, preconception counselling in RA women should cover this topic, too, helping women who are considering pregnancy understand the importance of not postponing conception too much. Preconception evaluation should focus not only on disease activity and RA treatment but also on identifying systemic involvement and comorbidities that might impact the outcome or need specialised evaluation and treatment, especially from organ-threatening involvement like lung, heart, or kidney disease [41]. Cardiopulmonary X-ray, pulmonary functional tests, and heart and abdominal ultrasound will help identify systemic involvement.

#### 3.2.4. Autoantibody with Impact on Pregnancy or Foetal Outcome

Although not specific to RA, testing for anti-Ro/SSA and anti-La/SSB antibodies should be done only once for women with RA during the preconception evaluation and not repeated during pregnancy, as recommended by the 2020 American College of Rheumatology guideline for the management of reproductive health in Rheumatic and Musculoskeletal diseases, as their presence is associated with the risk of foetal heart block and neonatal lupus, especially with a moderate or high titre [21]. The risk of congenital heart block (CHB) is around 2% in primigravid women, and the recurrence rate in future pregnancy is 6–10 times higher [43]. There is no consensus about foetal surveillance, but the ACR guideline recommends foetal echocardiograms and properly informing future mothers about the risk and the monitoring process [21]. The Preventive Approach to Congenital Heart Block with Hydroxychloroquine (PATCH) trial recommends starting Hydroxychloroquine (HCQ) 400 mg/day no later than 10 weeks into the pregnancy to lower the risk of CHB [43]. HCQ is not as widely used in RA as in Systemic Lupus Erythematosus (SLE), and at preconception counselling, the physician should discuss the importance of starting HCQ as soon as pregnancy is confirmed in RA women at risk [21].

Evaluation for thyroid disease is mandatory in any future mother irrespective of RA diagnosis. Anti-Ro antibodies are associated with an increased risk of CHB in the presence of hypothyroidism [44], suggesting the need for stringent monitorisation and compliance to HCQ.

Less frequently, women with RA have associated antiphospholipid antibodies: lupus anticoagulant, anti-cardiolipin, or β2 glycoprotein 1(β2GP1) antibodies. Screening for them in case of different clinical manifestations, such as thrombotic events [micro or macrovascular] or past pregnancy morbidity, will lower future pregnancy risks [45].

#### 3.2.5. Joint Involvement with Anaesthetic Impact: Cervical Subluxation, Cricoarytenoid, and Temporomandibular Joint Involvement

Specific joint involvements, including cervical disco vertebral, cricoarytenoid, and temporomandibular joints, may impose greater difficulty during an anaesthesiology procedure. Cervical involvement with atlantoaxial subluxation and subsequent neurologic damage is due to C1–C2 instability, and the risk is increased by prolonged glucocorticoid use, seropositivity, the presence of rheumatoid nodules, and erosive peripheral disease. Screening with lateral cervical radiography with flexion and extension view during the preconception period is necessary for RA women with cervical pain if at high risk [46,47]. Cricoarytenoid joint disease and temporomandibular arthritis may trigger difficult intubation or airway obstruction after extubating, of which both the anaesthesiologist and the RA patient must be aware [48].

#### 3.2.6. Medication

Women with RA should plan a pregnancy when their disease is in remission or LDA for at least 6 months with the help of medication considered safe during pregnancy [21,37]. Not long ago, not only future mothers with RA but also treating physicians thought that the natural evolution of RA was going into remission during pregnancy, thus embracing the tendency to lower or completely stop their medication during the nine months of gravidity [32]. Recently, the Pre CARA study showed the beneficial effect of a T2T approach in women pursuing pregnancy and during pregnancy using allowed medication (Sulfasalazine and/or Hydroxychloroquine as the first line and adding less than 7.5 mg/day Prednisone and/or TNF inhibitors as the next step) [37]. The study demonstrated that ACR recommendations for pursuing pregnancy during stable remission or LDA are perfectly attainable, allowing for a safe pregnancy.

Assessing disease activity and medication in women with RA during preconception counselling is mandatory as the objective is to plan pregnancy during stable, inactive, or LDA under accepted medication [49]. Changing to pregnancy-safe treatment should be done in advance of conception, allowing the patient and the medical team to observe and treat signs of flares before pregnancy. Women with RA should be reassured that if flares occur during pregnancy, there are accepted medications that can be used for a proper outcome for both the mother and the baby.

Both EULAR and ACR have made recommendations about how to use medication in AID before and during pregnancy and during lactation, helping clinicians to make advised decisions in daily life situations [20,21]. These recommendations were updated in 2024 by EULAR taking into account the accumulated safety data. According to this, all anti TNFα can be used throughout pregnancy, representing a valid option in the preconception period to obtain and maintain proper disease control. Other biological treatments, such as anti TNF α-Rituximab, Tocilizumab, Sarilumab, and Abatacept, might represent an option to control the mother’s active disease during pregnancy if other options fail/are contraindicated (second line indication). JAK inhibitors (Baricitinib, Tofacitinib, Upadacitinib, Filgotinib), a new, oral option treatment for RA, have insufficient safety data on use during pregnancy and should be avoided until more data become available, so it is advisable to switch to an approved medication before conception [50]. Table 2 emphasises how RA medication should be used in the preconception period from the pregnancy safety point of view.

### 3.3. Practical Approach for Preconception Counselling and Care Pathway in Women with RA 

As they have a chronic illness, women with RA with child-bearing potential should undergo periodic preconception counselling to avoid unplanned pregnancy. Starting with the moment of diagnosis and regularly after that, rheumatologists, general practitioners, and obstetricians should discuss family planning and contraception with their patients. This will contribute to a better future pregnancy outcome with no supplemental risk related to disease activity, forbidden medication, or no diagnosed or treated comorbidities or extra-skeletal RA manifestation. Table 3 summarises preconception counselling from the RA perspective.

Patients with RA who are pursuing pregnancy should be followed by a dedicated multidisciplinary team formed by a dedicated rheumatologist and an obstetrician with expertise in maternofoetal surveillance with the help of an anaesthesiologist or other speciality when needed. The beneficial effects of a multidisciplinary clinical pathway for pregnancy in different rheumatic conditions including RA are now available [28,49]. A reduced risk of adverse pregnancy outcomes with lower miscarriage/perinatal death was found in pregnancies followed by a dedicated team, a risk similar to the one for the general population [28].

**Figure 2:**
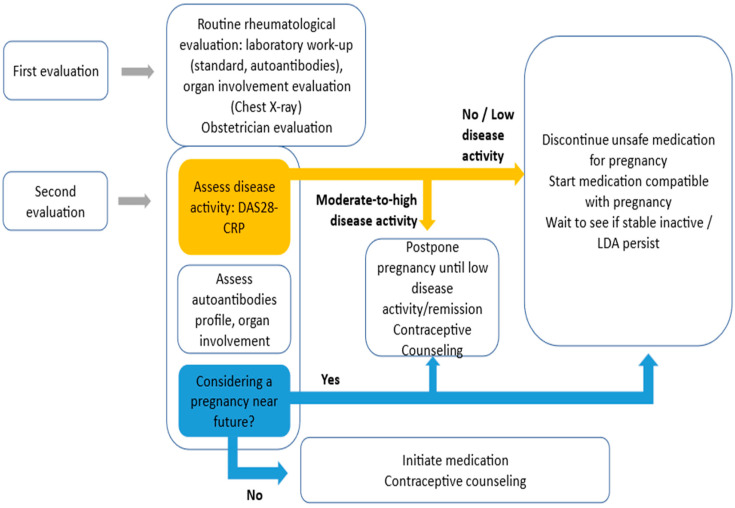
Preconception evaluation of reproductive-age women with RA.

Figure 2 illustrates the proposed multidisciplinary team approach during preconception evaluation in RA women. Patients should be evaluated periodically during the preconception phase until the optimal time for conception—a stable, inactive disease with proper medication. Contraceptive counselling and physiological support should be offered when pregnancy is not advised, reassessing the case at regular intervals after that.

## 4. Conclusions

Nowadays, pregnancy is increasingly common in AID due to advancements in the management of this category of patients. Still, implementation of a preconception pathway involving a team formed by a rheumatologist, an obstetrician, and a general practitioner is frequently lacking in clinical practice. These could help improve not only care for patients but also expand knowledge that is so much needed. Sharing experiences should be able to help accumulate data for stronger recommendations in a field that is based mostly on registries or indirect results from trials or observational studies. This is available not only for the medical team, but also for patients, who should now be part of shared decision making. All of them should have access to the latest knowledge in the field in order to make the best decision.

Biological treatment represents an important step forward for these patients, helping to achieve the target or to maintain it, giving women with RA the possibility of a normal family life. Overall, a general rule is to aim for at least six months of inactive or low disease before conception under permitted medication. Medication adjustments in anticipation of pregnancy according to T2T principles and accepted medication and a dedicated team with expertise in following AID pregnancy will contribute to achieving a similar risk rate in RA pregnancy compared to the general population.

Unmet educational needs remain a challenge for everyone, including patients and doctors, and active interventions are necessary to expand effective educational resources and increase the quality of care in the most precious time for a woman.

## Figures and Tables

**Table 1:** Characterisation of RA activity according to DAS 28-ESR and DAS 28-CRP.

Disease Status—ESR	Disease Status—CRP	DAS 28 Value
<2.6	<2.4	Disease remission
2.6–3.2	2.4–2.9	Low disease activity
3.2–5.1	2.9–4.6	Moderate disease activity
>5.1	>4.6	High disease activity

**Table 2:** Managing medication during the preconception period in women with RA [12,20,21,43,50].

Medication	Action During Preconception
Sulfasalazine	Continue
Hydroxychloroquine	Continue. In patients with anti-Ro, anti-La positive should be added, preferably before 10 weeks gestation
Prednisone	Can be used if needed, preferably less than 7.5 mg/day
NSAID [preferably not cyclooxygenase 2 inhibitors]	Can be used. Stop if woman is having difficulty conceiving
TNF α inhibitors	Continue (all TNF inhibitors can be used during pregnancy). Certolizumab might be used throughout the pregnancy, while Infliximab, Adalimumab, Etanercept, and Golimumab should be stopped during the 3rd trimester
Methotrexate	Discontinue 1–3 months prior to conception
Leflunomide	Discontinue for 3.5 months and check serum metabolite before conception; use cholestyramine for wash-out
Rituximab, Tocilzumab, Abatacept	May be used only if needed during pregnancy to control active disease of the mother; better to switch to an approved medication before conception
JAK inhibitors (Baricitinib, Tofacitinib, Upadacitinib, Filgotinib)	Insufficient safety data on use during pregnancy; discontinue before conception and switch to an approved therapy

**Table 3:** Steps to perform during preconception counselling for RA women.

Evaluation for Preconception Counselling from the RA Perspective
Clinical evaluation: anamnesis [important to find out if the patient needed to increase or to add Prednisone or immunosuppressive therapy in the preceding 6 months for active disease] and complete physical exam
Lab evaluation: complete blood count, inflammatory markers, urinalysis, renal and hepatic function, glucose level, coagulation tests, thyroid function evaluation, including for autoimmune thyroiditis
Immune markers associated with RA: RF, ACPA Immune markers with prognostic implications: ANA, anti-Ro, anti-La antibodies, antiphospholipid antibodies
Disease activity evaluation: perform DAS 28 score
Assessment of extra-skeletal organ involvement with the specific investigation [ultrasound, pulmonary function, imaging if required]
Assessment of skeletal involvement with anaesthesiology implications [atlantoaxial subluxation, cricoarytenoid, or temporomandibular joint involvement]
Assessment of medication considering the T2T principle and pregnancy-approved medication
Assessment for frequent comorbidities in women with RA with impact on pregnancy outcome: hypertension, renal failure, diabetes mellitus, thrombosis
Assessment of reproductive history: past fertility or pregnancy history
General principle: assess risk for congenital infection, thyroid function, and vaccination status; supplement with Vitamin D and Folic Acid
